# Supplementary material for: Caspase-1 cleaves PPARγ for potentiating the pro-tumor action of TAMs
Source: Nat Commun. 2017 Oct 3;8:766. doi: 10.1038/s41467-017-00523-6 (PMC5626701; doi:10.1038/s41467-017-00523-6)
Supplement: Supplementary file 1 — Supplementary Information [file 41467_2017_523_MOESM1_ESM.pdf]

Description of Supplementary Files

File Name: Supplementary Information
Description: Supplementary Figures, Supplementary Tables and Supplementary Methods

File Name: Supplementary Data 1
Description: Genes associated with caspase-1 cleavage: genes whose expression patterns changed after treatment with caspase-1 inhibitors, for example, genes up-regulated in the coculture group (Coculture vs Ctrl) but down-regulated upon administration of YVAD (YVAD vs Coculture), or vice versa. Using a cutoff threshold of a 1.5-fold change in expression and a false discovery rate threshold of 0.05, we selected 618 genes for analysis.

File Name: Supplementary Data 2
Description: Pathway classification analysis on the 618 pre-selected genes.

File Name: Supplementary Data 3
Description: Gene Ontology analysis of the differentially expressed genes associated with metabolism in TAM-like cells.

File Name: Supplementary Data 4
Description: Pathway analysis of the differentially expressed genes associated with metabolism in TAM-like cells.

File Name: Peer Review File

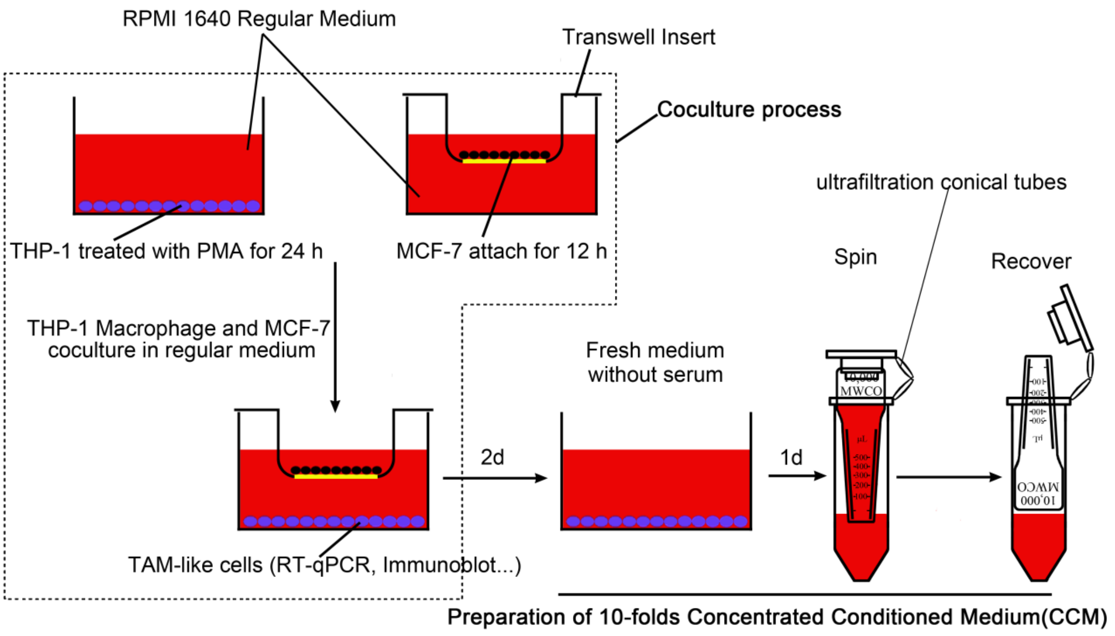

Supplementary Figure 1. Schematic of the coculture model. The coculture procedure is depicted in the circle marked with dotted lines. Briefly, 2 mL THP-1 cells (1×10^5 cells/mL) were seeded in 6-well plates and treated with 50 nM PMA for 24 h to induce macrophage differentiation. The PMA-containing medium was discarded and the cells were washed 3 times in PBS to remove residual PMA. PMA-treated THP-1 macrophages (in 6-well plates) were cocultured with 1 mL MCF-7 (1×10^5 cells/mL, and MCF-7 has been incubated with the upper inserts for 12 h to attach before coculture) in fresh RPMI1640 supplemented with 10% FBS and 1% penicillin-streptomycin for the indicated periods of time.

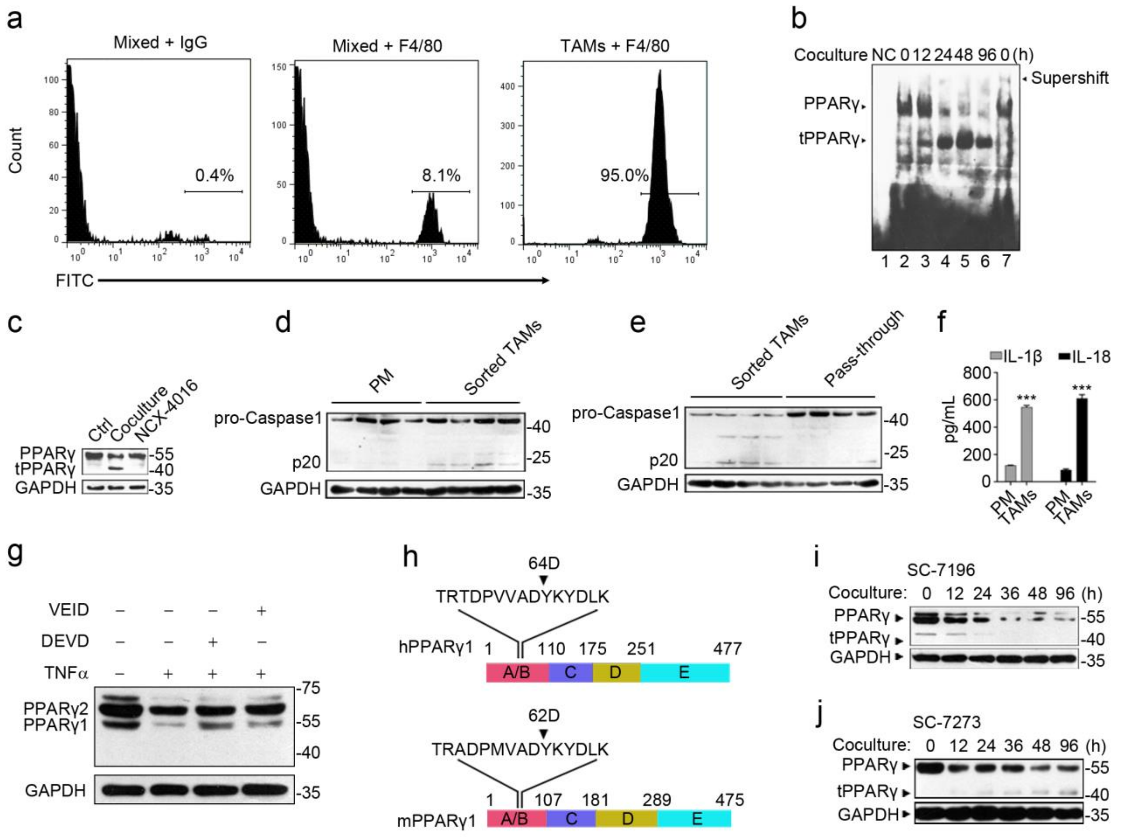

Supplementary Figure 2. Caspase-1 mediates PPARγ cleavage at Asp64 in TAMs both *in vitro* and *in vivo.* (**a**) (left to right) Flow cytometry analysis FITC intensity in mixed tumor tissue cells incubated with IgG isotype control, mixed tumor tissue cells incubated with anti-F4/80 antibody, TAMs incubated with anti-F4/80 antibody. (**b**) PPARγ and tPPARγ in nuclear fractions at indicated time points was analyzed by EMSA. (**c**) THP-1 macrophages were cultured alone (Ctrl) or co-cultured with MCF-7 in the absence (Coculture) or presence of NCX-4016 (NCX-4016), and then PPARγ and its truncated fragment were determined by immunoblot analysis. (**d**,**e**) Pro-caspase-1 and it's cleaved active forms in sorted TAMs and peritoneal macrophages (PM) (**d**), sorted TAMs and pass-through cells (**e**) from MMTV-PyVT tumor bearing mice were determined by immunoblot analysis. (**f**) The releasing of IL-1β and IL-18 from peritoneal macrophage (PM) and sorted TAMs from MMTV-PyVT tumor bearing mice were measured by ELISA. The data represent means ± s.e.m., (n =8, ***P<0.001, Student's *t*-test). (**g**) 3T3-L1 adipocytes were treated with 25 ng ml^{-1} TNFα overnight in the presence or absence of caspase-3 inhibitor

DEVD (50 μM) or caspase-6 inhibitor VEID (50 μM), lysed, and the PPARγ protein analyzed by western blot, using a carboxyl-terminal anti-PPARγ monoclonal antibody (clone E-8) (**h**) Diagram showed the conserved cleavage region on human and mouse PPARγ. (**i**,**j**) THP-1 macrophages were co-cultured with MCF-7 for indicated time points, and PPARγ and its truncated fragment were analyzed by immunoblot with the antibody (sc-7273) recognizing the C-terminal of PPARγ (**i**) or the antibody (sc-7196) recognizing the N-terminal of PPARγ (**j**).

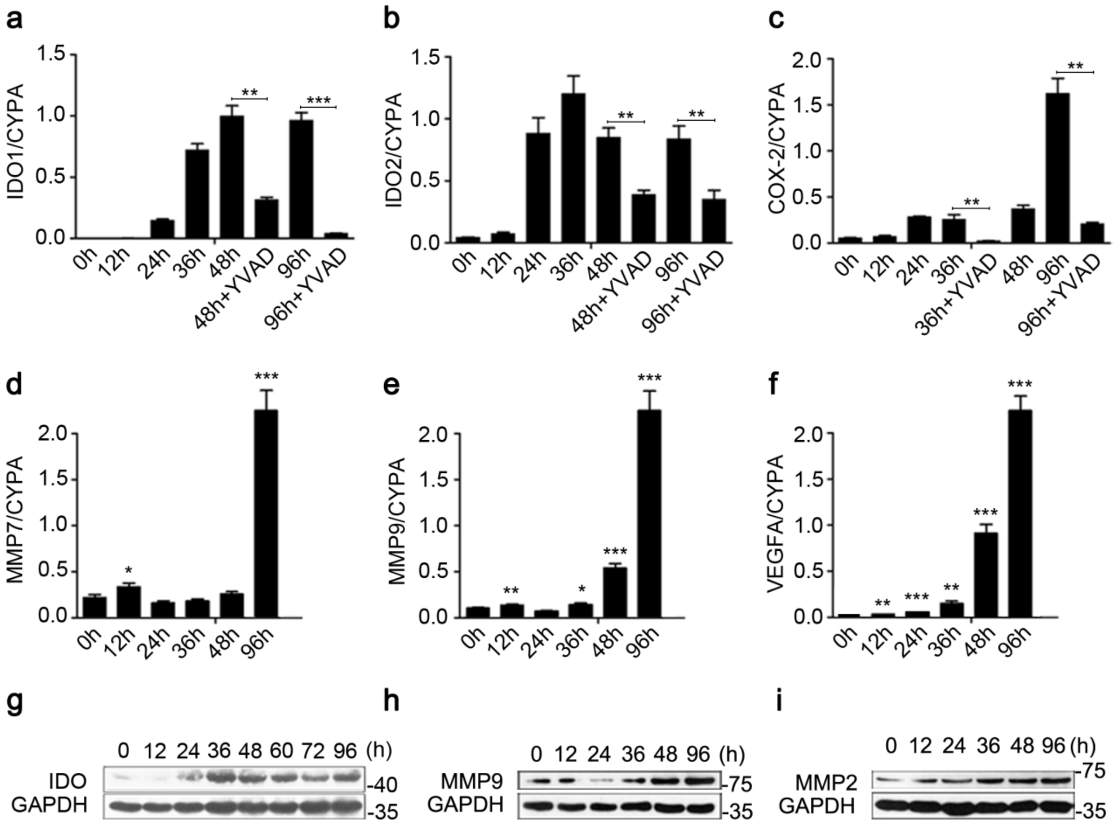

Supplementary Figure 3. Pro-tumoral genes or proteins are up-regulated after PPARγ cleavage. (**a-f**) THP-1 macrophages were co-cultured with MCF-7 for indicated time points in the presence or absence of caspase-1 inhibitor YVAD, and then mRNA expression of indicated genes was analyzed by RT-qPCR**.** (**g-i**) The expression of IDO, MMP9 and MMP2 proteins were analyzed by immunoblot**.** All the histograms in this figure show means ± s.e.m., (n =3, *P<0.05, **P<0.01, ***P<0.001, Student's *t*-test).

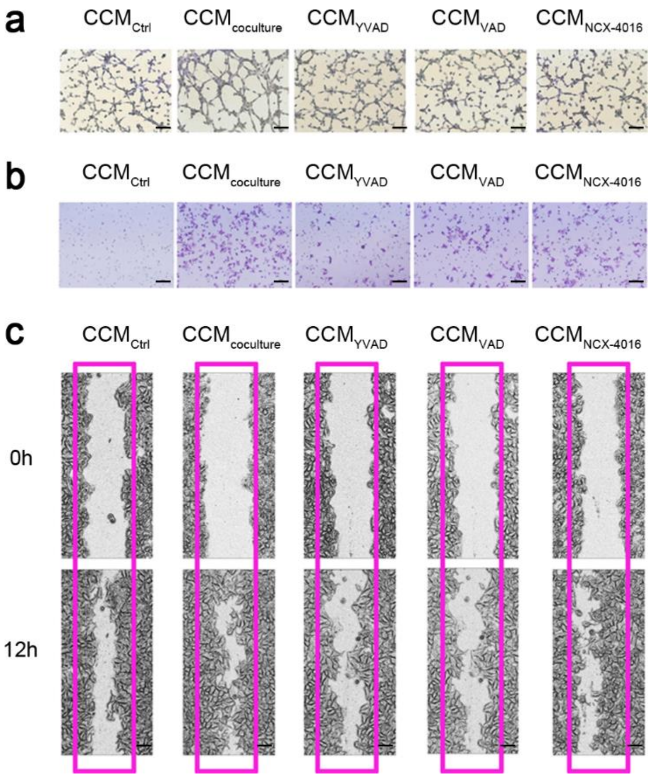

Supplementary Figure 4. Caspase-1 inhibition impairs the pro-tumoral abilities of TAMs. (**a**) For angiogenesis assay, HUVEC were preincubated with CCM_{Ctrl}, $CCM_{Coculture}$, CCM_{YVAD}, CCM_{VAD} and $CCM_{NCX-4016}$ for 12 h. The cells were then seeded in 24-well plates coated with Matrigel (1×10^5 cells/well). After 6 h of culture at 37°C, representative photographs were taken, scale bar 100 µm, and the average number of tubules was calculated and analyzed. (**b**) For invasion assay, MCF-7 cells were seeded into Matrigel-coated invasion chambers and incubated with CCM_{Ctrl}, $CCM_{Coculture}$, CCM_{YVAD}, CCM_{VAD} and $CCM_{NCX-4016}$ for 12 h, MCF-7 cells were fixed, stained with crystal violet, then representative photographs were taken, scale bar 100 µm, and invaded cells were counted and analyzed. (**c**) For scratch migration assay, MCF-7 cells grown to 90% confluency monolayers were incubated in the absence of serum for 12 h and wounded in a line across the well with a 10 µl standard pipette tip. The wounded monolayers were washed twice with serum free media to remove cell debris and then incubated with CCM_{Ctrl}, $CCM_{Coculture}$, CCM_{YVAD}, CCM_{VAD} and $CCM_{NCX-4016}$ for another 12 h. Representative photographs were taken, scale bar 100 µm, migrated cells were counted and analyzed. All Photographs were taken with an Nikon

DXM1200C microscope equipped with digital camera.

*The quantitative data were generated from the mean value of three independent experiments, mean value of each experiment was based on at least 10 fields.

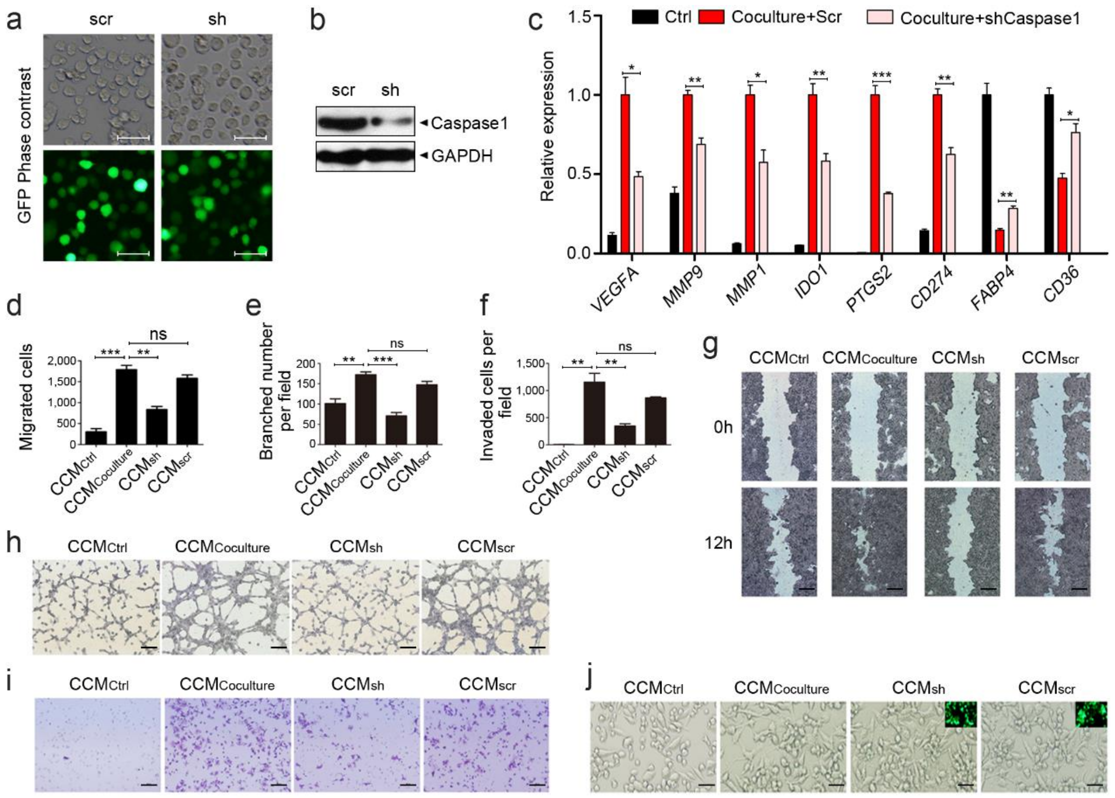

Supplementary Figure 5. Caspase-1 knockdown impairs the pro-tumoral abilities of TAMs.

(**a**) Phase contrast and fluorescence microscopy of THP-1 cells stably expressing shRNA against caspase-1 (sh) or control sramble shRNA (scr) sorted by FACS, scale bar 50 µm. (**b**) Caspase-1 expression in THP-1 cells stably expressing shRNA against caspase-1 (sh) or control sramble shRNA (scr) sorted by FACS was measured by immunoblot. (**c**) THP-1_{sh} and THP-1_{scr} were cultured alone or cocultured with MCF-7 for 48 h, the expression of protumoral genes were assessed by RT-qPCR. (**d,g**) For scratch migration assay, MCF-7 cells were grown to 90% confluency monolayers were incubated in the absence of serum for 12 h and wounded in a line across the well with a 10 ml standard pipette tip. The wounded monolayers were washed twice with serum free media to remove cell debris and then incubated with CCM_{Ctrl}, $CCM_{Coculture}$, CCM_{Sh} and CCM_{Scr} for another 12 h. Migrated cells were counted and analyzed. Representative photographs were taken, scale bar 100 µm. (**e,h**) For invasion assay, MCF-7 cells were seeded into Matrigel-coated invasion chambers and incubated with CCM_{Ctrl}, $CCM_{Coculture}$, CCM_{Sh} and

CCM_{Scr} for 12 h, MCF-7 cells were fixed, stained with crystal violet. Migrated cells were counted and analyzed. Representative photographs were taken, scale bar 100 μm. (**f,i**) For angiogenesis assay, HUVEC were pre-incubated with CCM_{Ctrl}, $CCM_{Coculture}$, CCM_{Sh} and CCM_{Scr} for 12 h. The cells were then seeded in 24-well plates coated with Matrigel (1×10^5 cells/well). After 6 h of culture at 37°C. Average number of tubules was counted and analyzed. Representative photographs were taken, scale bar 100 μm. (**j**) Pictures of macrophage cocultured with MCF-7 for 96 h were taken. All Photographs were taken with an Nikon DXM1200C microscope equipped with digital camera, scale bar 100 μm. All the histograms in this figure show means ± s.e.m., (n =3, *P<0.05, **P<0.01, ***P<0.001, ns, not significant, Student's *t*-test).

***d,e,f** The quantitative data were generated from the mean value of three independent experiments, mean value of each experiment was based on at least 10 fields.

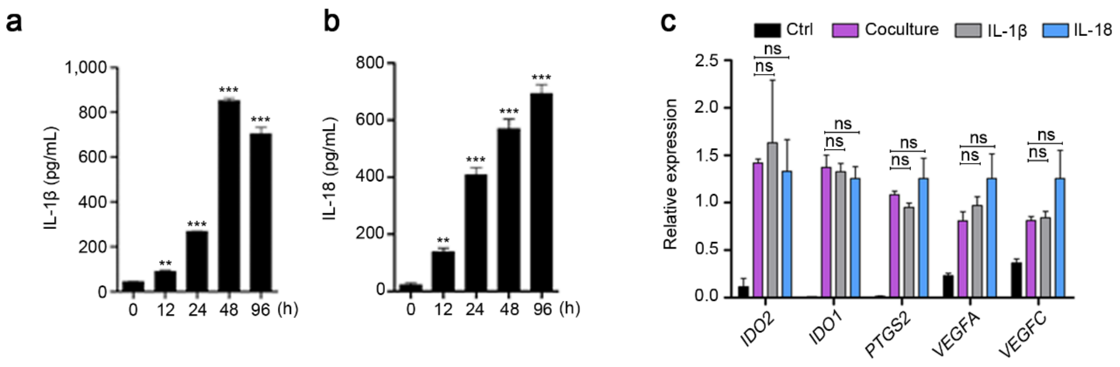

Supplementary Figure 6. Caspase-1 substrates IL-1β and IL-18 are not involved in TAM differentiation. (**a**,**b**) The IL-1β (**a**) and IL-18 (**b**) levels of cell culture supernatant in coculture system were measured by ELISA at indicated time points. (**c**) THP-1 cells were cultured alone or cocultured with MCF-7 or cocultured with MCF-7 in the presence of 600 pg ml^{-1} IL-1β or 600 pg ml^{-1} IL-18, the expression of pro-tumoral genes were assessed by RT-qPCR. All the histograms in this figure show means ± s.e.m., (n =3, **P<0.01, ***P<0.001, ns, not significant, onw way ANOVA).

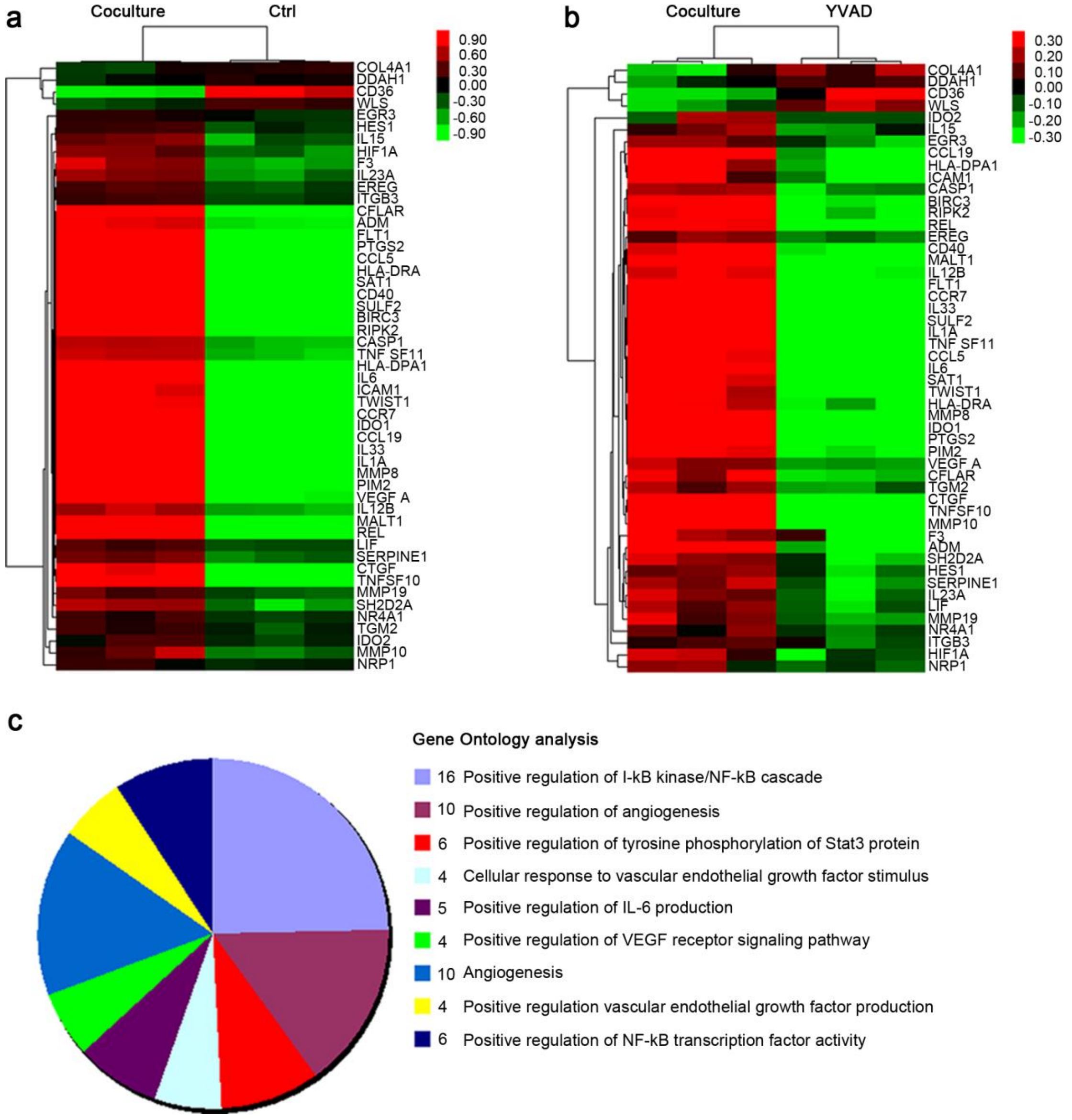

Supplementary Figure 7. Microarray analysis of pro-tumoral genes expression during THP-1 differentiation. THP-1 macrophages cultured alone (Ctrl) or co-cultured with MCF-7 for 48 h in the absence (Coculture) or presence of caspase-1 inhibitors YVAD (YVAD) were subjected to microarray analysis. Genes whose expression patterns can be reversed by YVAD are selected. (**a**,**b**) Heat map of 33 genes (p-value < 0.05, fold change > 1.5 or < -1.5) closely linked with pro-tumoral functions of macrophage are presented. (**c**) Gene ontology analysis performed on genes that are differentially expressed in (**a**,**b**).

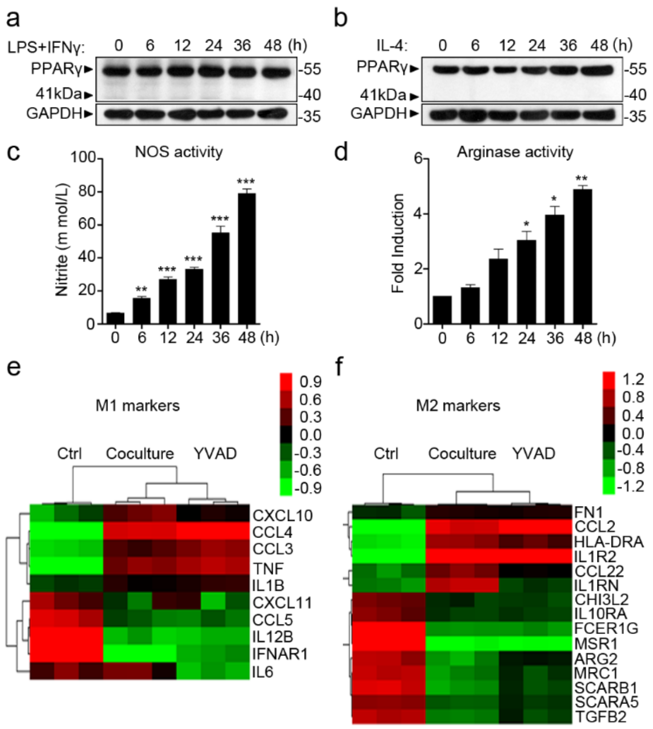

Supplementary Figure 8. Caspase-1 cleaves PPARγ doesn't involve in the differentiation of M1 or M2. (a,c) THP-1 macrophages were incubated for the indicated time with 20 ng/mL IFNγ and 100 ng/mL LPS, extracts of THP-1 cells were harvested at indicated time, PPARγ and its truncated fragment in the polarization of M1 was determined by immunoblot analysis **(a)**, NO production in the culture supernatant was measured by griess method **(c)**. **(b,d)** Cells were incubated for the indicated times with 10 ng/mL IL-4, extracts of THP-1 cells were harvested at indicated time, PPARγ and its truncated fragment in the polarization of M2 was determined by immunoblot analysis **(b)**, relative arginase activity was measured by arginase activity assay kit **(d)**. **(e,f)** Microarray analysis of M1 **(e)** or M2 **(f)** specific genes expression in THP-1 macrophages cultured alone or cocultured with MCF-7 in the absence or presence of YVAD. All the histograms in this figure show means ± s.e.m., (n =3, *P<0.05, **P<0.01, ***P<0.001, Student's *t*-test).

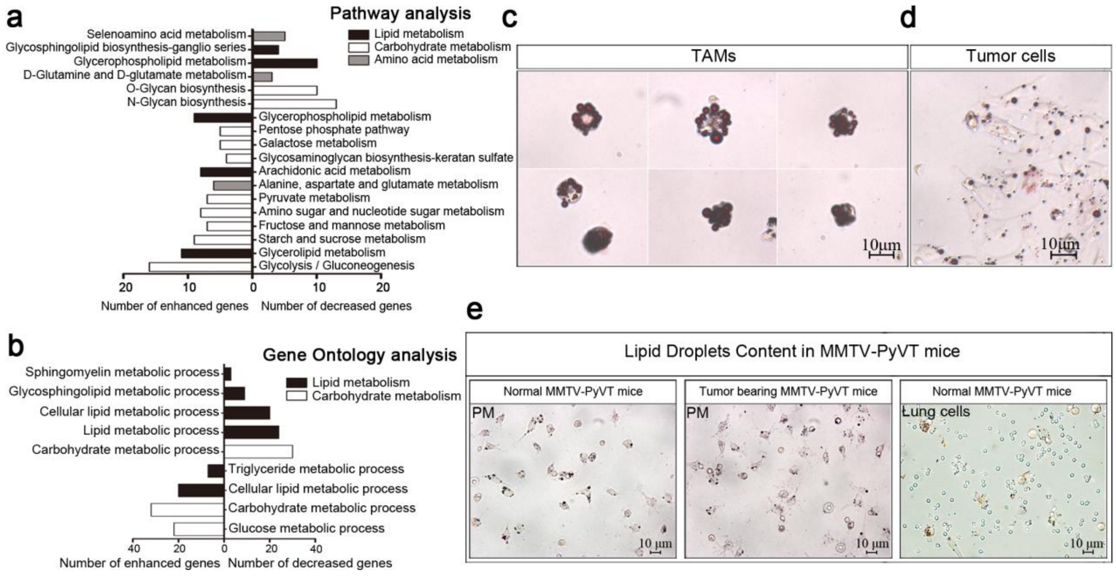

Supplementary Figure 9. Metabolic reprogramming of TAMs. (**a**,**b**) THP-1 macrophages cultured alone (Ctrl) or co-cultured with MCF-7 for 48 h (Coculture) were subjected to microarray analysis, genes that related to metabolism are pre-selected (p-value < 0.05, fold change > 1.5 or < -1.5), Pathway analysis (**a**) and gene ontology analysis (**b**) were performed for these genes. (**c**) Lipid content was analyzed by Oil Red O staining in TAMs isolated from tumors from MMTV-PyVT mice. (**d**) Lipid content was analyzed by Oil Red O staining in tumor cells from MMTV-PyVT mice. (**e**) Lipid content was analyzed by Oil Red O staining in peritoneal macrophages isolated from tumor bearing MMTV-PyVT mice or normal MMTV-PyVT mice and single cell suspensions from normal MMTV-PyVT mice lung tissues.

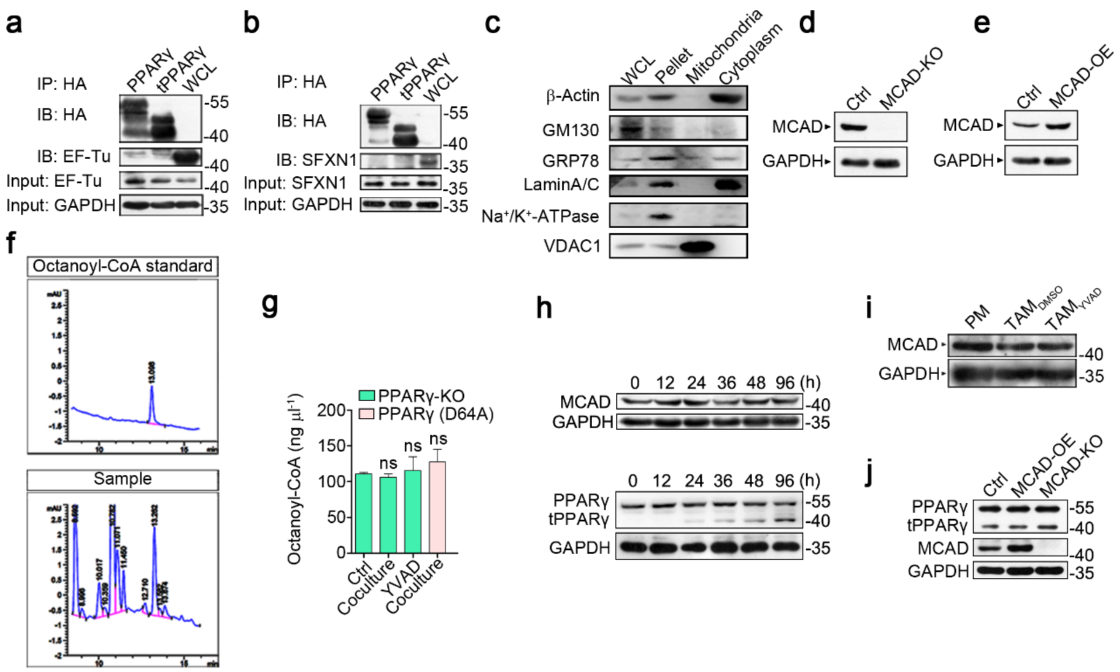

Supplementary Figure 10. tPPARγ enriches in mitochondria and binds to MCAD then impairs its activity.

(**a**) THP-1 cells were transfected with plasmids encoding HA-tagged full-length PPARγ or tPPARγΔ64D. Cell extracts were prepared 48 h post-transfection and immunoprecipitated with anti-HA antibodies, then, immunoblot analysis of coimmunoprecipitated proteins with anti-HA antibodies for two forms of PPARγ or anti-EF-Tu antibodies for detection of EF-Tu. (**b**) THP-1 cells were transfected with plasmids encoding HA-tagged full-length PPARγ or tPPARγΔ64D. After 48 h, immunoprecipitated with anti-HA antibodies, then, immunoblot analysis of coimmunoprecipitated proteins with anti-HA antibodies for two forms of PPARγ or anti-SFXN1 antibodies for detection of SFXN1. (**c**) Mitochondria purity was analyzed by immunoblotting with antibodies against Na^+,K^+-ATPase β2 (a plasma membrane marker), GM130 (a Golgi marker), LaminA/C (a nucleus marker), voltage-dependent anion channel (VDAC, a mitochondrial outer membrane marker), β-actin (a cytoplasm marker) and Bip/GRP78 (an endoplasmic reticulum marker). (**d**,**e**) Immunoblot analysis of MCAD knockout and overexpression efficiency in THP-1 cells. (**f**) Retention time (13 min) was confirmed by using purified Octanoyl-CoA standard. HPLC analyzes the retention time of purified octanoyl-coA standard (upper), and

components of one sample (lower). (**g**) PPARγ knockout THP-1 macrophages were cultured alone or cocultured with MCF-7 in the absence or presence of YVAD for 24 h, PPARγ (D64A) overexpressing THP-1 macrophages (wild type PPARγ was deleted) were cocultured with MCF-7 for 24 h, then MCAD activity was analyzed by HPLC. Remaining Octanoyl coenzyme A were negatively correlated with MCAD activity. Statistical results of total three trials was showed. The data represent means ± s.e.m., (n =3, ns, not significant, Student's *t*-test). (**h**) THP-1 macrophages were cocultured with MCF-7 for indicated time, then MCAD protein, PPARγ and PPARγ fragment in THP-1 macrophages were measured by immunoblot. (**i**) Peritoneal macrophages were isolated from tumor bearing MMTV-PyVT mice, and TAMs were isolated from tumor bearing MMTV-PyVT mice administration of vehicle or caspase-1 inhibitor YVAD, MACD expression was analyzed by immunoblot. TAMs in each sample were pooled from three mice. (**j**) PPARγ and tPPARγ expression in wild type, MCAD knockout and MCAD overexpression THP-1 macrophages cocultured with MCF-7 for 24 h were analyzed by immunoblot.

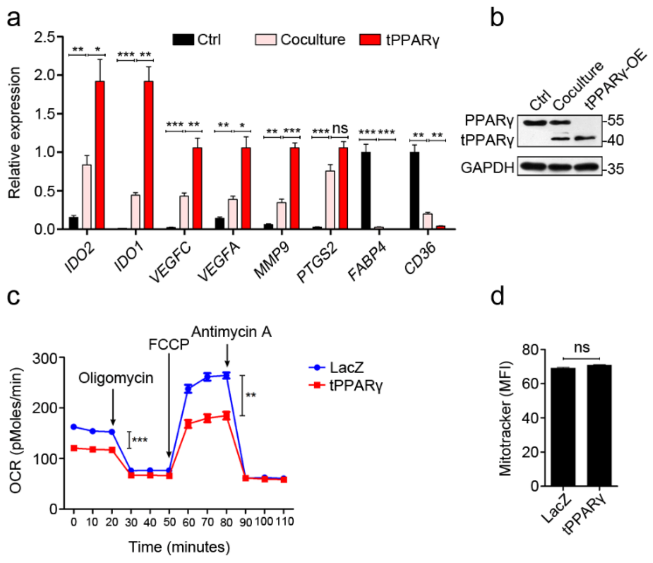

Supplementary Figure 11. tPPARγ overexpression promotes TAM differentiation.

(**a**) LacZ overexpressing THP-1 macrophages were cultured alone (Ctrl), or cocultured with MCF-7 for 2 d (Coculture), tPPARγ overexpressing THP-1 macrophages (wild type PPARγ was deleted) were cocultured with MCF-7 for 2 d, TAMs hallmarks were analyzed by RT-qPCR. (**b**) Immunoblot analysis of the tPPARγ overexpression efficiency. (**c**) The oxygen consumption rates (OCRs) in were measured in overexpressing LacZ control THP-1 or tPPARγ-expressing macrophages at baseline and in response to mitochondrial inhibitors, as specified in the Experimental Procedures. (**d**) Mitochondria content in these macrophages was measured by mitotracker. All the histograms in this figure show means ± s.e.m., (n =3, *P<0.05, **P<0.01, ***P<0.001, ns, not significant, Student's *t*-test).

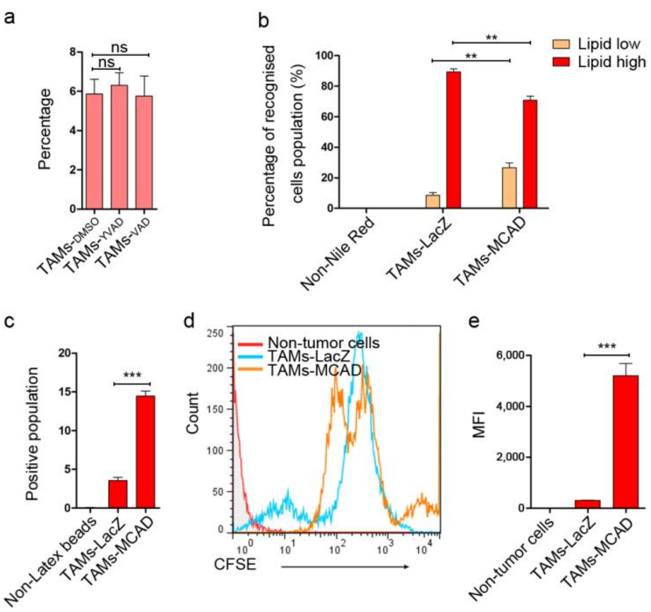

Supplementary Figure 12. tPPARγ overexpression promotes TAM differentiation.

(**a**) MMTV-PyMT tumor bearing mice were administrated with vehicle, YVAD or VAD for 3 weeks, TAMs were sorted from the total tumor tissue cells by magnetic cell sorting using MACS, and TAMs percentage in mixed cells was analyzed (n=6). (**b**) Statistics of **Fig. 9j** were presented. Statistics were generated based on three trials (n=7). (**c**) Statistics of **Fig. 9l** were presented. Statistics were generated based on three trials (n=7). (**d,e**) Phagocytosis of 4T1 tumor cells by TAMs-LacZ and TAMs-MCAD was analyzed by flow cytometry (**d**). And statistics of three trials were showed (**e**), the phagocytic index mean fluorescence intensity (MFI) was generated by FlowJo software (n=7). All data represent means ± s.e.m., (**P<0.01, ***P<0.001, ns, not significant on way ANOVA).

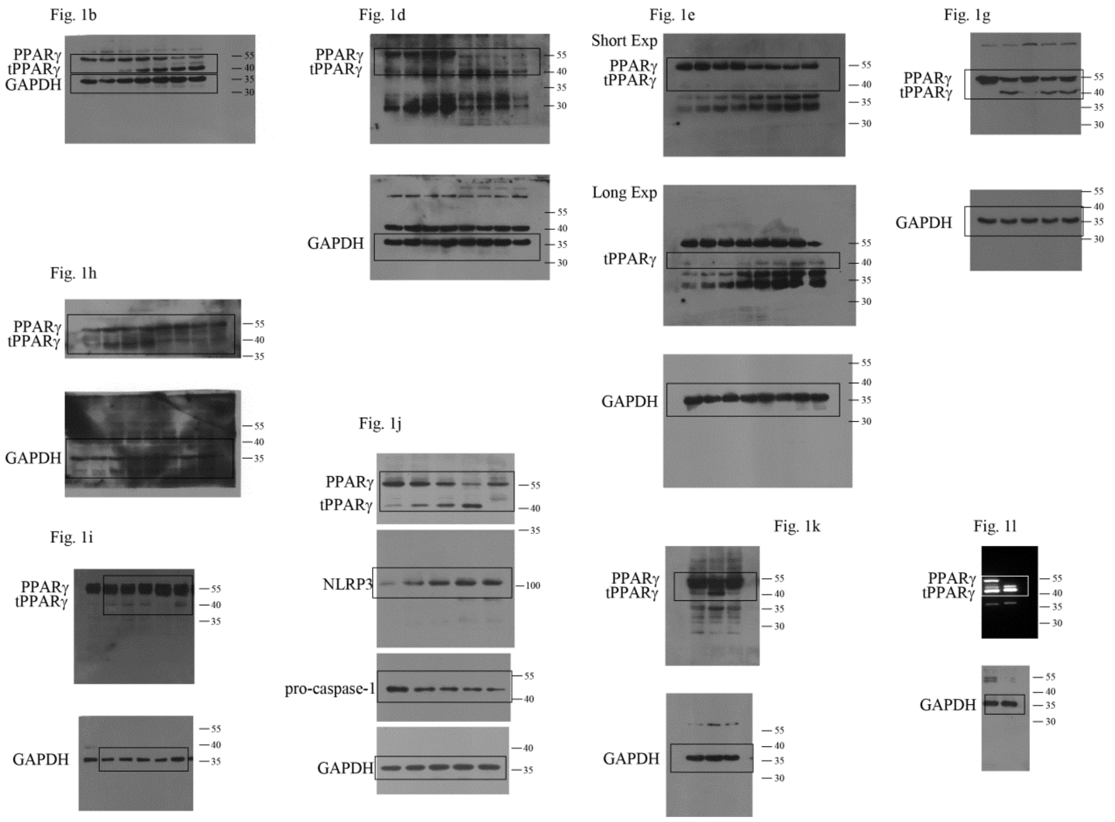

Supplementary Figure 13: full scans of blots in Figure 1b, Figure 1d, Figure 1e, Figure 1g, Figure 1h, Figure 1i, Figure 1j, Figure 1k and Figure 1l

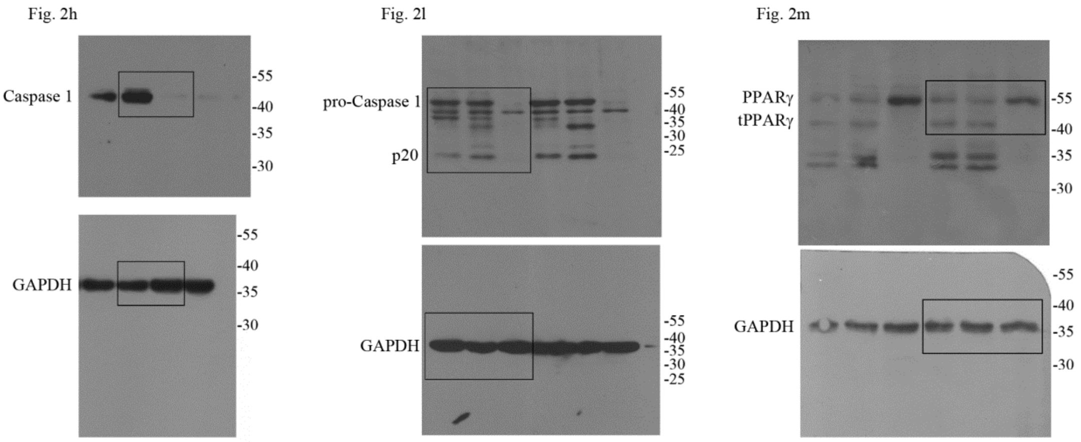

Supplementary Figure 14: full scans of blots in Figure 2h, Figure 2l and Figure 2m

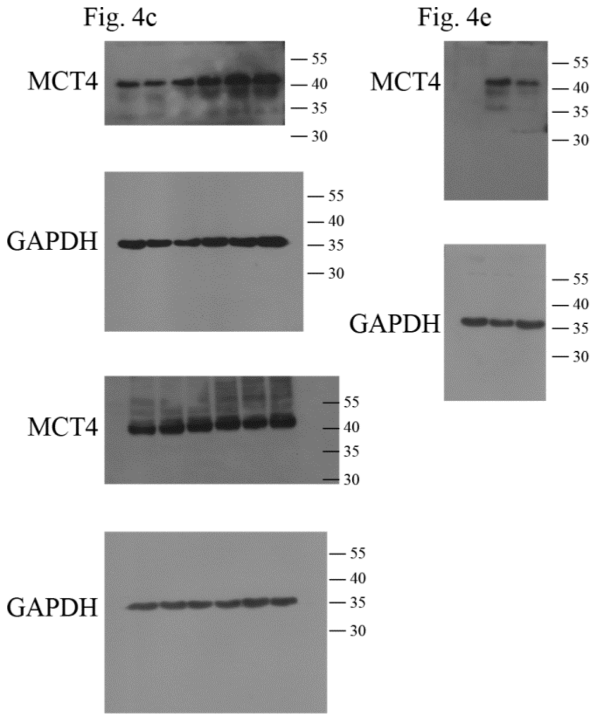

Supplementary Figure 15: full scans of blots in Figure 4c, Figure 4e

Fig. 5d

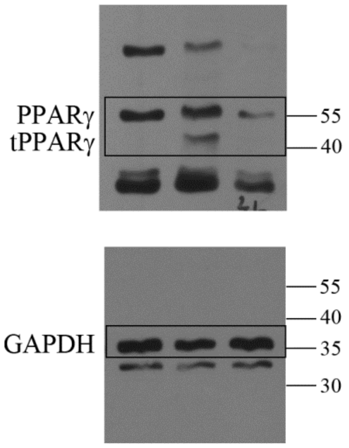

Fig. 5h

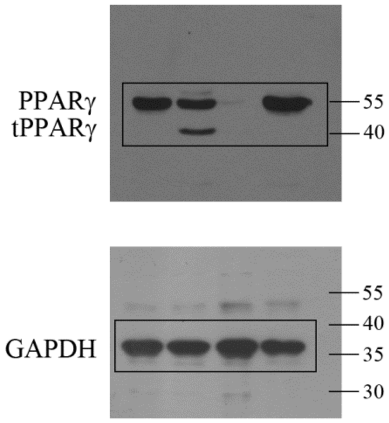

Supplementary Figure 16: full scans of blots in Figure 5d, Figure 5h

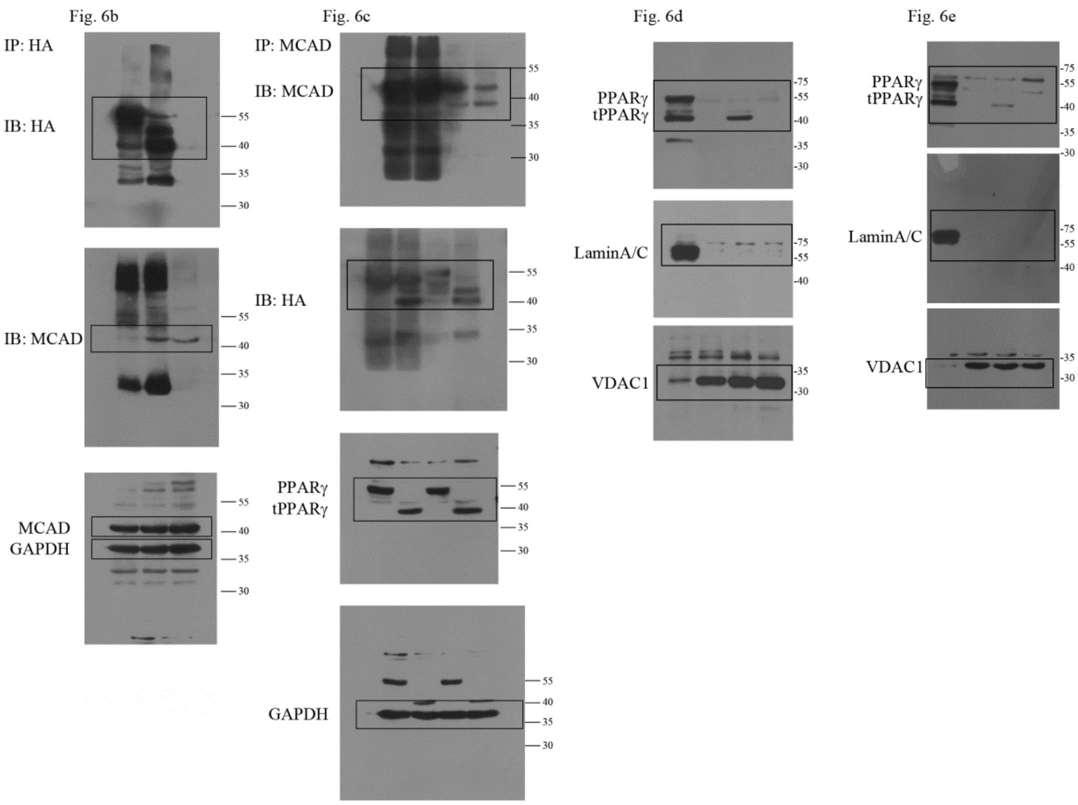

Supplementary Figure 17: full scans of blots in Figure 6b, Figure 6c, Figure 6d and Figure 6e

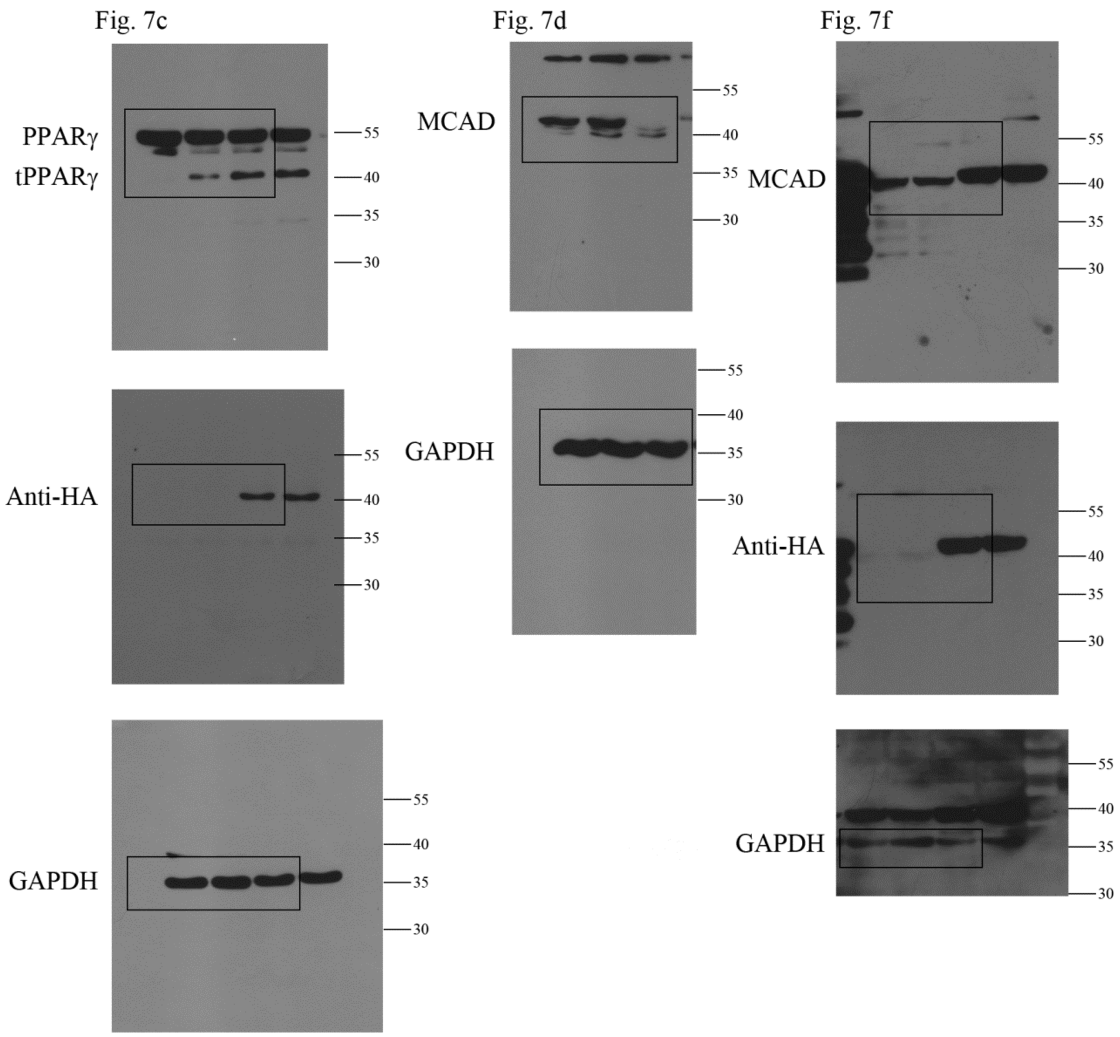

Supplementary Figure 18: full scans of blots in Figure 7c, Figure 7d and Figure 7f

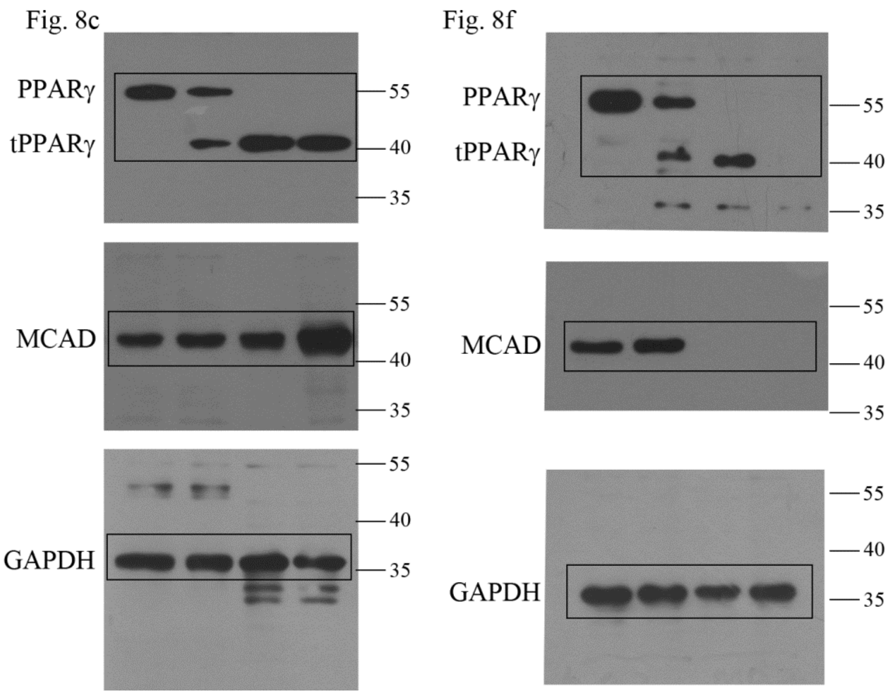

Supplementary Figure 19: full scans of blots in Figure 8c and Figure 8f

Fig. 9i

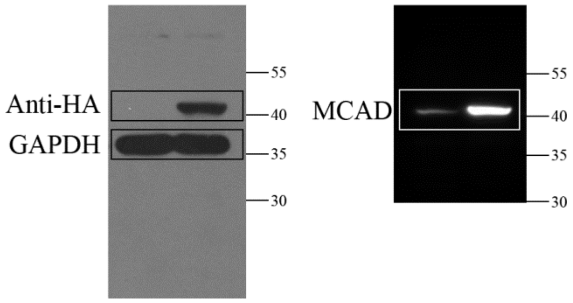

Supplementary Figure 20: full scans of blots in Figure 9i

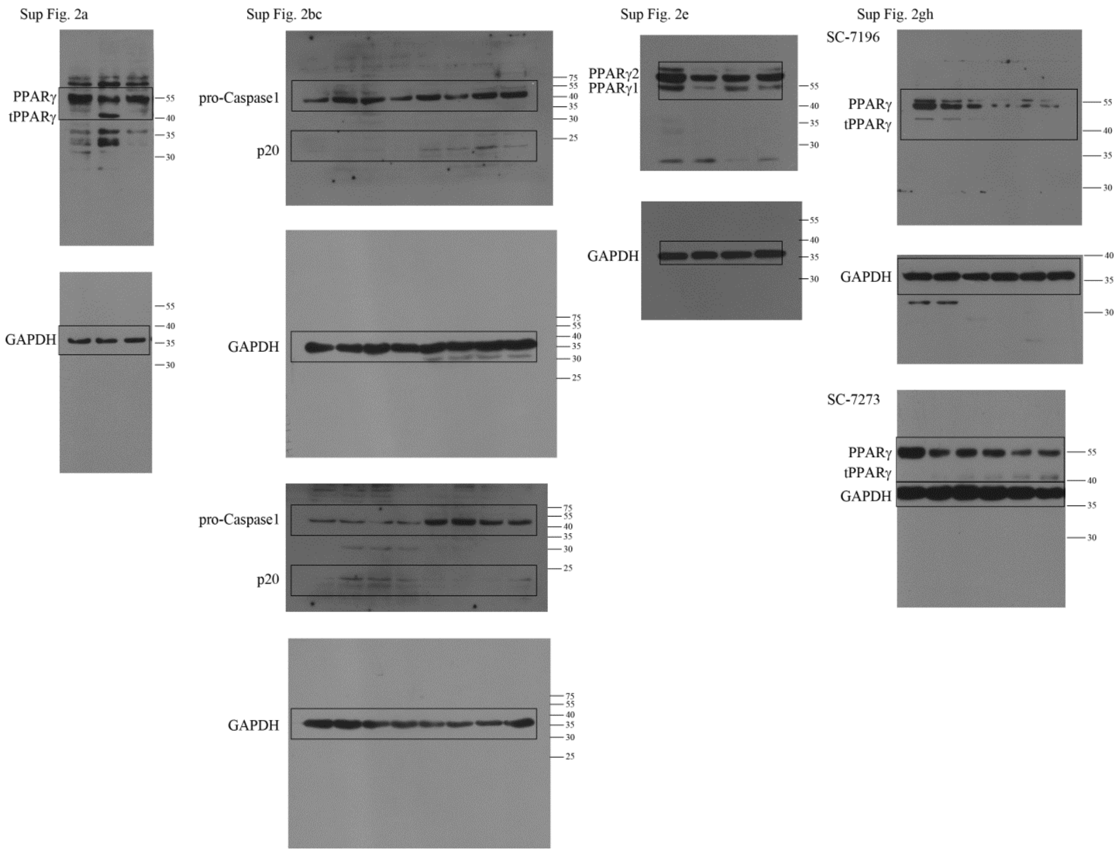

Supplementary Figure 21: full scans of blots in Supplementary Figure 2a, Supplementary Figure 2bc, Supplementary Figure 2e and Supplementary Figure 2gh

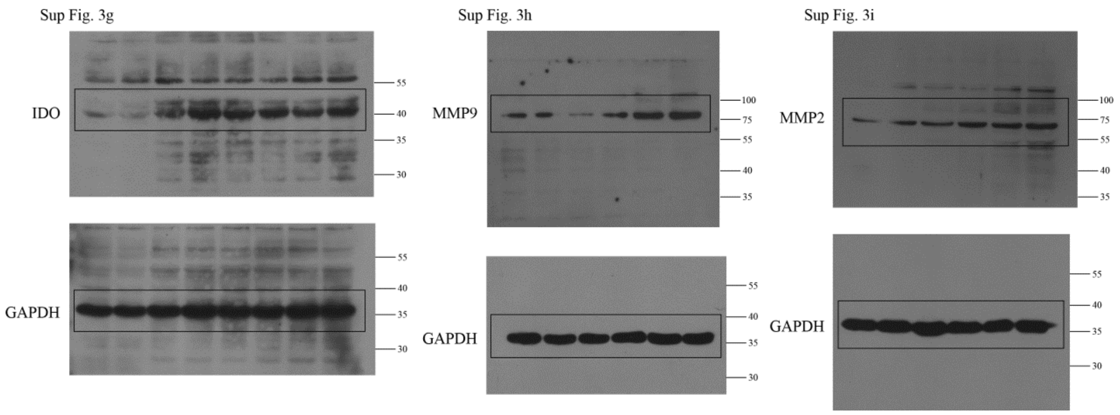

Supplementary Figure 22: full scans of blots in Supplementary Figure 3g, Supplementary Figure 3h and Supplementary Figure 3i

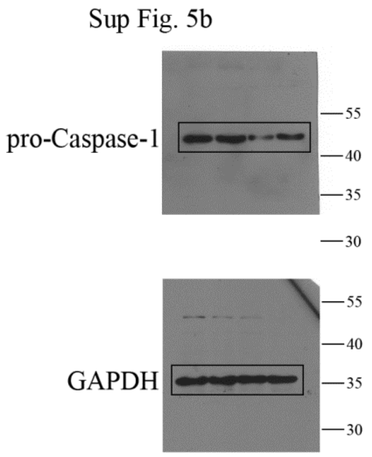

Supplementary Figure 23: full scans of blots in Supplementary Figure 5b

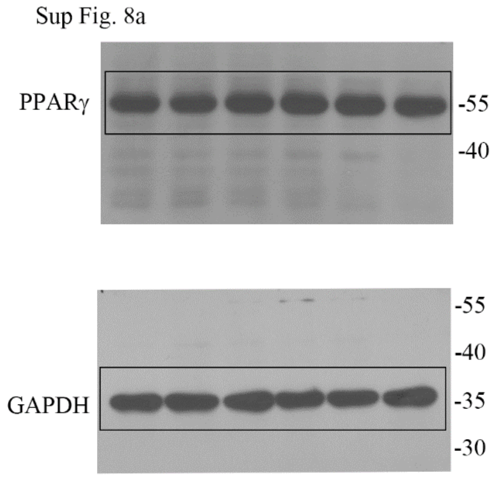

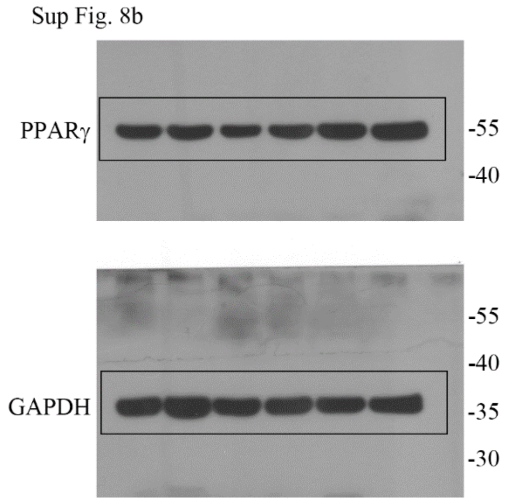

Supplementary Figure 24: full scans of blots in Supplementary Figure 8a and Supplementary Figure 8b

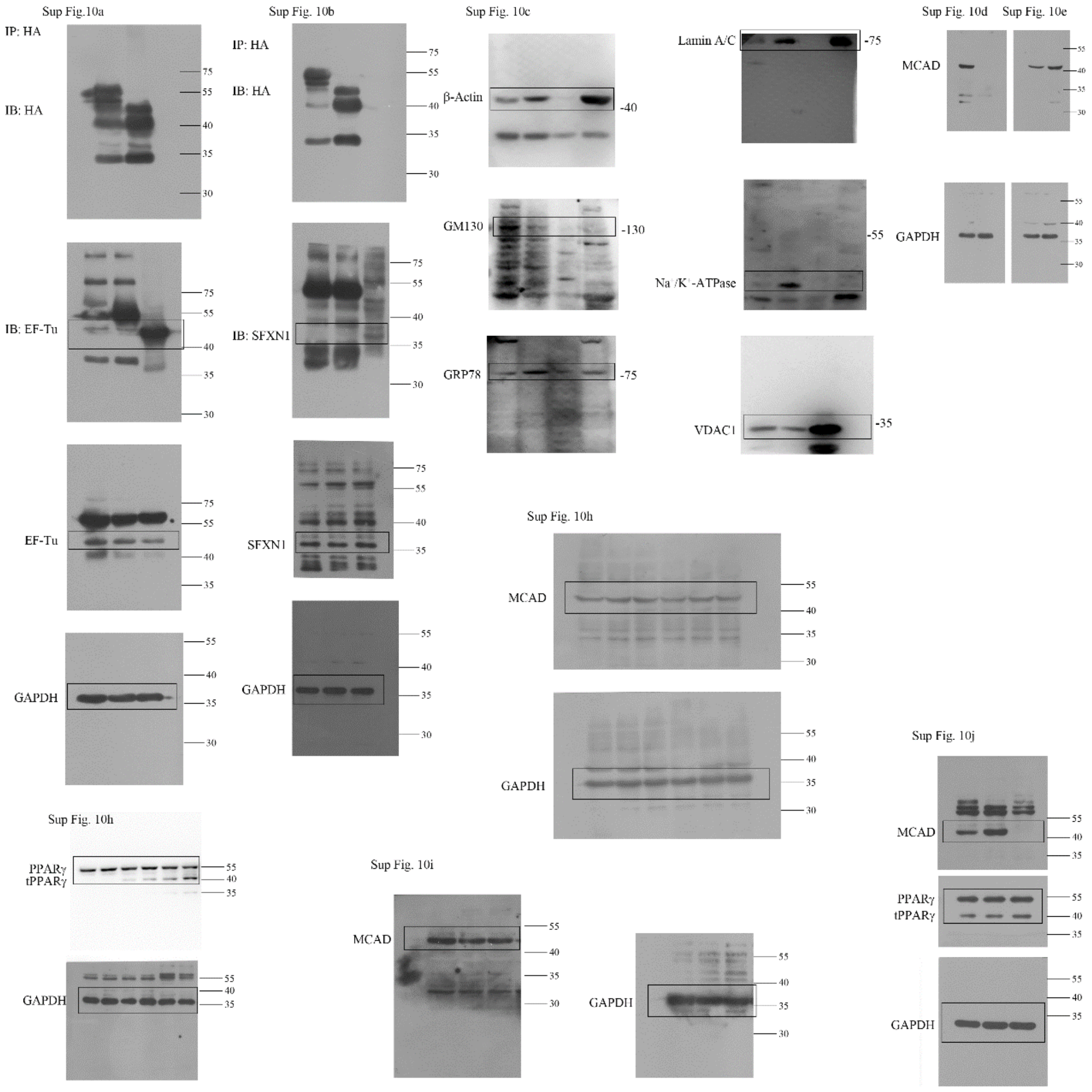

Supplementary Figure 25: full scans of blots in Supplementary Figure 10a, Supplementary Figure 10b, Supplementary Figure 10c, Supplementary Figure 10d, Supplementary Figure 10e, Supplementary Figure 10h, Supplementary Figure 10i and Supplementary Figure 10j

Sup Fig. 11b

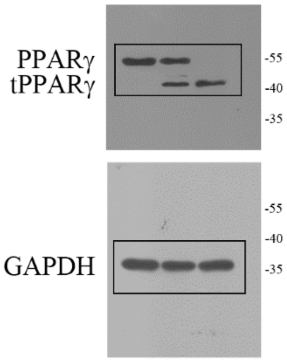

Supplementary Figure 26: full scans of blots in Supplementary Figure 11b

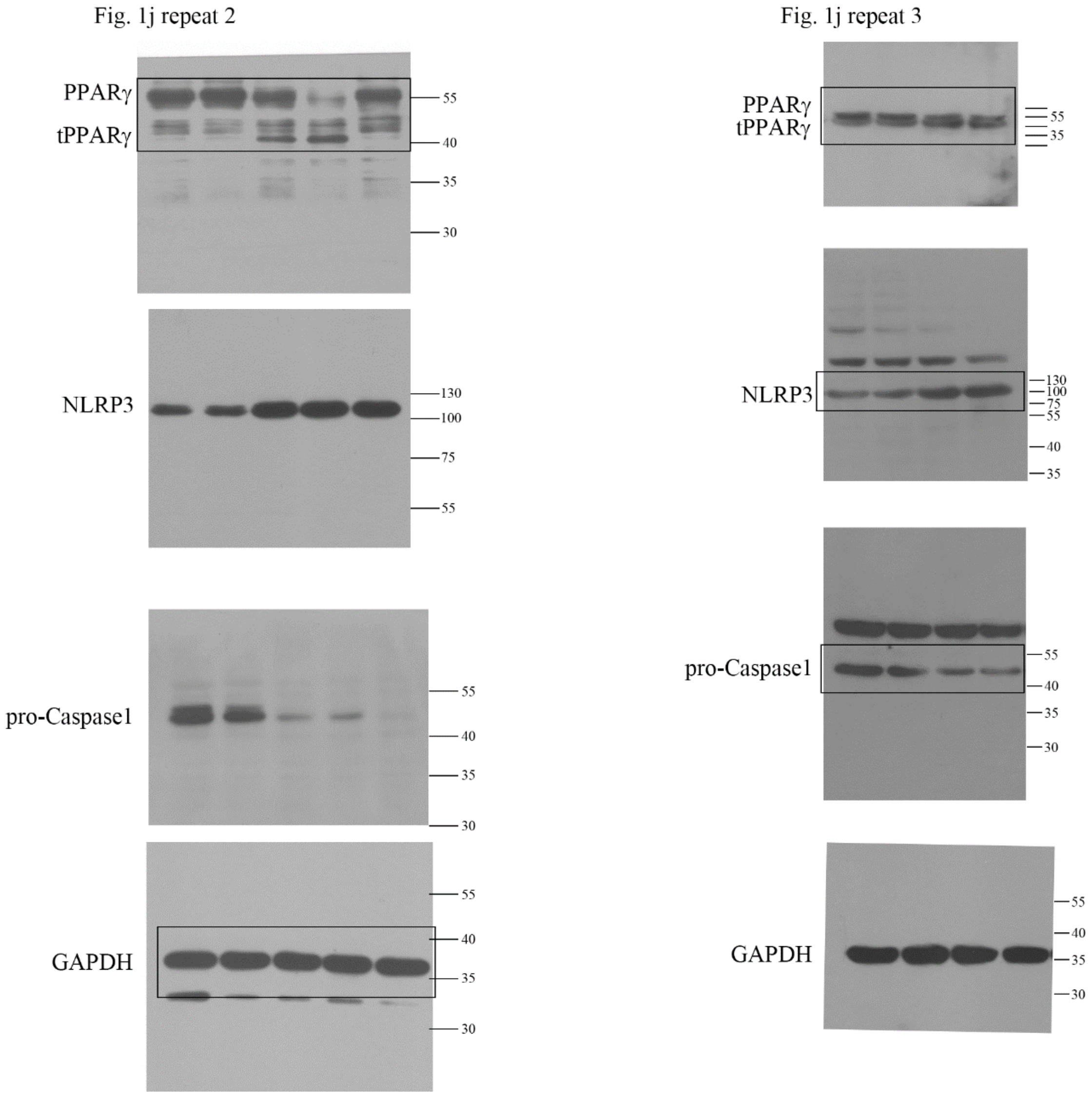

Supplementary Figure 27: full scans of blots in Figure 1j(another two repeats)

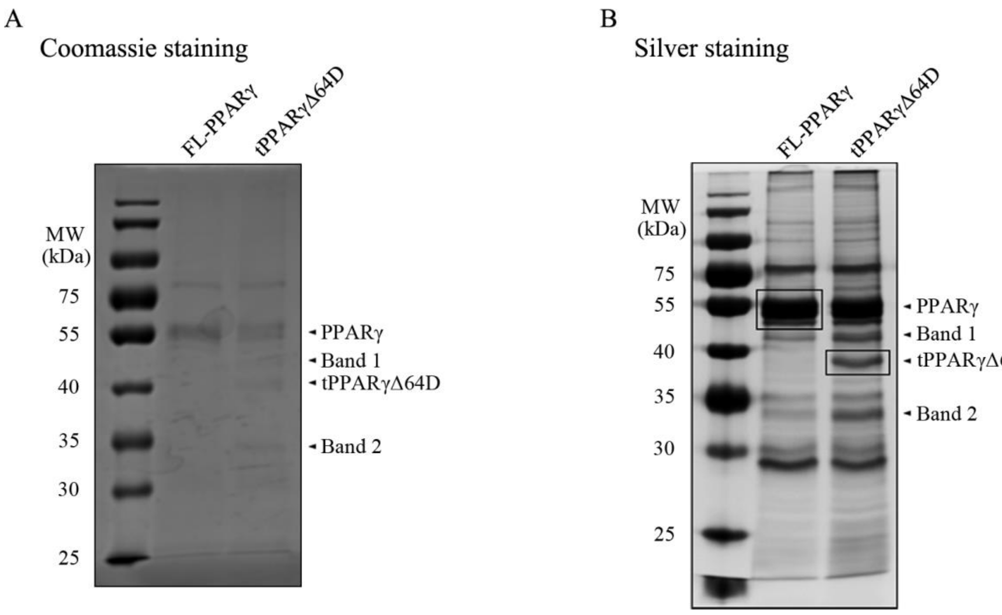

Supplementary Figure 28: Coomassie and silver staining of Figure 6a

**Supplementary Table 1. Silver staining shows examples of the potential cellular proteins that specifically interact with tPPARγΔ64D.**				
Swiss-Prot accession	Identified proteins	Score	No. of Unique Peptides	Molecular weight (kDa)
Band 1				
P37231-2	Isoform 1 of PPARγ	347.58	22	54.6
P60709	Actin, cytoplasmic 1	132.24	17	41.7
P04264	Keratin, type II cytoskeletal 1	119.75	17	66.0
P13645	Keratin, type I cytoskeletal 10	92.19	13	58.8
P35908	Keratin, type II cytoskeletal 2	84.42	13	65.4
P39023	60S ribosomal protein L3	81.69	16	46.1
Q03181-2	Isoform 2 of PPARδ	74.77	1	40.4
P35527	Keratin, type I cytoskeletal 9	59.11	7	62.0
P49411	Elongation factor Tu	56.28	10	49.5
B7Z9I1	MCAD	55.47	6	42.4
P38646	Stress-70 protein,	51.88	8	73.6
Band 2				
P37231-2	Isoform 1 of PPARγ	581.61	14	54.6
P63244	GNB2L1	101.08	12	35.1
P13645	Keratin, type I cytoskeletal 10	93.68	14	58.8
P04264	Keratin, type II cytoskeletal 1	88.71	14	66.0
Q9Y6D9	Mitotic spindle assembly checkpoint protein	84.20	12	83.0
Q07955	Serine/arginine-rich splicing factor 1	81.46	9	27.7
P35527	Keratin, type I cytoskeletal 9	56.78	9	62.0
P62424	60S ribosomal protein L7a	55.86	8	30.0
Q9H9B4	Sideroflexin-1	53.25	9	35.6
P02647	Apolipoprotein A-I	51.03	11	30.8

**Supplementary Table 2. Primers used in this article.**			
Human		Mouse	
Names	Sequences	Names	Sequences
MMP9(F)	GAACCAATCTCACCGACAGG	*B7h1*(F)	GCTCCAAAGGACTTGTACGTG
MMP9(R)	GCCACCCGAGTGTAACCATA	*B7h1*(R)	TGATCTGAAGGGCAGCATTTC
VEGFA(F)	AAGGAGGAGGGCAGAATCAT	*Ptgs2*(F)	TTCCAATCCATGTCAAAACCGT
VEGFA(R)	TTCTTGCGCTTTCGTTTTT	*Ptgs2*(R)	AGTCCGGGTACAGTCACACTT
VEGFC(F)	CTACAGATGTGGGGGTTGCT	*Ido1*(F)	TGGCGTATGTGTGGAACCG
VEGFC(R)	CATCCAGCTCCTTGTTTGGT	*Ido1*(R)	CTCGCAGTAGGGAACAGCAA
MMP1(F)	CTGGCCACAACTGCCAAATG	*Ido2*(F)	TCAAAGTCAGAGCATGACGCT
MMP1(R)	CTGTCCCTGAACAGCCCAGTACTTA	*Ido2*(R)	GGCGGTTCTCGATTAAGTGAG
IDO1(F)	GCCAGCTTCGAGAAAGAGTTG	*Vegfa*(F)	GCACATAGAGAGAATGAGCTTCC
IDO1(R)	ATCCCAGAACTAGACGTGCAA	*Vegfa*(R)	CTCCGCTCTGAACAAGGCT
IDO2(F)	CCACAGACCGAATGTGAAGAC	*B7h4*(F)	ATCATTGGCTTTGGCATTTC
IDO2(R)	TGTTGGCAATTTCCATCCAAGG	*B7h4*(R)	TCAGGTTCAAAAGTGCAGCTC
PTGS2(F)	CTGGCGCTCAGCCATACAG	*Folr2*(F)	CGAGGACAAGCTGCATGA
PTGS2(R)	CGCACTTATACTGGTCAAATCCC	*Folr2*(R)	GGGAGTCAGCCTTGTGTAGC
CD36(F)	AGTCACTGCGACATGATTAATGGT	*Mmp2*(F)	TAACCTGGATGCCGTCGT
CD36(R)	CTGCAATACCTGGCTTTTCTC	*Mmp2*(R)	TTCAGGTAATAAGCACCCTTGAA
FABP4(F)	TACTGGGCCAGGAATTTGAC	*Mmp9*(F)	ACGACATAGACGGCATCCA
FABP4(R)	GTGGAAGTGACGCCTTTCAT	*Mmp9*(R)	GCTGTGGTTCAGTTGTGGTG
PPARγ(F)	AACCACCCTGAGTCCTCAC	*Arg1*(F)	TGGCTTGCGAGACGTAGAC
PPARγ(R)	TCATGTCTGTCTCCGTCTT	*Arg1*(R)	GCTCAGGTGAATCGGCCTTTT
PPIA(F)	GCATACGGGTCCTGGCATCTTGTCC	*Chil3*(F)	TTATCCTGAGTGACCCTTCTAAG
PPIA(R)	ATGGTGATCTTCTTGCTGGTCTTGC	*Chil3*(R)	TCATTACCCTGATAGGCATAGG
		Ppia(F)	GCATACGGGTCCTGGCATCTTGTCC
		Ppia(R)	ATGGTGATCTTCTTGCTGGTCTTGC

Supplementary Methods

Metabolism Assay. Oxygen consumption rates (OCR) were measured under basal conditions and in response to 1 μM oligomycin, 0.75 μM fluorocarbonyl cyanide phenylhydrazone (FCCP), and 0.5 μM antimycin A (Sigma) with the XF-24 Extracellular Flux Analyzer (Seahorse Bioscience).

Nitric Oxide Measurement. Macrophage were cultured with LPS and IFN-γ for 0-48 h. Synthesis and release of NO by macrophages was determined by assaying culture supernatants for nitrite content. Briefly, 100 μL supernatant was reacted for 10 minutes at room temperature with an equal volume of Griess reagent [0.5% sulfanilamide and 0.05% N-(1-naphthyl) ethylenediamine dihydrochloride in 2.5% phosphoric acid]. The optical density at 570 nm was then determined on a microtiter plate reader.

Arginase assays. Cells were washed with PBS and lysed with 0.1% Triton X-100 containing protease inhibitor (Roche, Nutley, NJ). To activate Arg-1, buffer containing Tris-HCl (25 mM) and MnCl2 (10 mM) was added and heated to 56°C for 10 minutes. L-arginine (0.5 M; Sigma) was added, and the samples were heated for 1 hour at 37°C. The hydrolysis of arginine was stopped with 800 μL of an acid solution mixture (H2SO4:H3PO4:H2O, 1:3:7). The amount of urea produced was determined using 9% α-isonitrosopropriophenone and compared with a standard curve with absorbance measured at 540 nm.

Mitochondria isolation. Cell Mitochondria Isolation Kit was obtained from Thermo Scientific (Cat# 89874). Briefly, 10-30 of Dounce strokes were performed to rupture the cells in the Isolation Buffer provided by the kit. Then homogenate was transferred into a 2.0 ml Eppendorf tube. The homogenate was centrifuged at 1,000 g for 10 min, 4□ (pellet was saved for quality analysis). The supernatant was again transferred into a new tube and

centrifuged at 11,000 g for 10 min at 4℃ (supernatant was saved for quality analysis). The pellet (mitochondria fraction) was collected and freeze at -80℃ for later use.
